# Viral Hyperparasitism in Bat Ectoparasites: Implications for Pathogen Maintenance and Transmission

**DOI:** 10.3390/microorganisms10061230

**Published:** 2022-06-16

**Authors:** Alexander Tendu, Alice Catherine Hughes, Nicolas Berthet, Gary Wong

**Affiliations:** 1 Viral Hemorrhagic Fevers Research Unit, CAS Key Laboratory of Molecular Virology and Immunology, Institute Pasteur of Shanghai, Chinese Academy of Sciences, Shanghai 200031, China; atvya@ips.ac.cn; 2 University of Chinese Academy of Sciences, Beijing 100049, China; 3 School of Biological Sciences, University of Hong Kong, Hong Kong SAR, China; achughes@hku.hk; 4 Unit of Discovery and Molecular Characterization of Pathogens, Centre for Microbes, Development, and Health, CAS Key Laboratory of Molecular Virology and Immunology, Institute Pasteur of Shanghai, Chinese Academy of Sciences, Shanghai 200031, China; nicolas.berthet@pasteur.fr; 5 Unité Environnement et Risque Infectieux, Cellule d’Intervention Biologique d’Urgence, Institut Pasteur, 75015 Paris, France

**Keywords:** ectoparasites, zoonotic spillover, arthropods, bat viruses, hyperparasitism

## Abstract

Humans continue to encroach on the habitats of wild animals, potentially bringing different species into contact that would not typically encounter each other under natural circumstances, and forcing them into stressful, suboptimal conditions. Stressors from unsustainable human land use changes are suspected to dramatically exacerbate the probability of zoonotic spillover of pathogens from their natural reservoir hosts to humans, both by increasing viral load (and shedding) and the interface between wildlife with livestock, pets and humans. Given their known role as reservoir hosts, bats continue to be investigated for their possible role as the origins of many viral outbreaks. However, the participation of bat-associated ectoparasites in the spread of potential pathogens requires further work to establish. Here, we conducted a comprehensive review of viruses, viral genes and other viral sequences obtained from bat ectoparasites from studies over the last four decades. This review summarizes research findings of the seven virus families in which these studies have been performed, including *Paramyxoviridae, Reoviridae, Flaviviridae, Peribunyaviridae, Nairoviridae, Rhabdoviridae* and *Filoviridae*. We highlight that bat ectoparasites, including dipterans and ticks, are often found to have medically important viruses and may have a role in the maintenance of these pathogens within bat populations.

## 1. Introduction

In March 2022, an outbreak of yellow fever virus (YFV) was reported in Isiolo, Kenya, with a case fatality rate of more than 11% [1]. This is the first report of YFV in Isiolo, as previous outbreaks were localized in other western counties of Kenya known for the nomadic lifestyles of local residents. YFV, along with dengue virus (DENV), Zika virus, Chikungunya virus, Powassan virus, Sindbis virus and West Nile virus (WNV), are categorized as arboviruses. Rather than a taxonomic grouping of relatedness, ‘arboviruses’ reflects their shared dependence on arthropod vectors for transmission. The involvement of *Culicinae* and *Anophelinae* mosquitoes in the transmission of the above-named viruses has been evaluated to varying degrees. However, the particular role played by parasitic arthropods requires further in-depth investigation, as typical behaviors may exacerbate the potential for the transfer of pathogens between individuals. In the USA, WNV caused 66 deaths out of 731 cases in 2020 alone [2]. For WNV, this represents the most recent of recurring annual reports of WNV incidence across all USA states. These occurrences highlight the threat posed by familiar arboviruses.

Birds of the order *Passeriformes* are considered the reservoir hosts for both WNV and Sindbis arboviruses, making them zoonoses [3]. Summarily, as for other zoonoses, this description highlights the suggestion that these two viruses, or their genetic predecessors, replicate naturally in non-human vertebrates within whom they acquire, by incidental mutations, the ability to infect and replicate in humans. Zoonotic infections, as exemplified by SARS-CoV-2, have the potential of causing more than 6.2 million deaths globally in less than three years [4]. Such zoonoses often involve intermediate hosts capable of enabling growth and transmission of the viral agents. As intermediate hosts, domestic mammals in sustained contact with humans have been demonstrated to be part of the transmission chain for viruses pathogenic to humans [5,6,7,8].

An exploratory study on arthropod-hosted viruses in China revealed that arthropods host most of the diversity present among negative-sense RNA viruses [9]. This is of particular importance because unlike other types of RNA viruses, these are not known to infect fungi, unicellular eukaryotes or bacteria [10]. Phylogenetically, monophyletic branches formed by the viral sequences described in the study did not cluster together but rather shared multiple nodes with different families of segmented and unsegmented vertebrate host-specific negative-sense RNA viruses. These included filoviruses, influenza viruses, hantaviruses, lyssaviruses, paramyxoviruses, arenaviruses and bornaviruses. Such a finding is consistent with the proposed polyphyletic origin of viruses [10]. This is because although the identified RNA viruses had similar genomic architectures, they each shared a more recent common ancestor with viruses in different families. Their shared genomic structures are therefore a result of convergence. This, together with the observation that bats are the largest chunk of mammalian rhabdovirus hosts [11], necessitate the investigation of bat-associated arthropods.

Over the last two decades, outbreaks of infectious diseases have occurred across all habitable continents with variable incidence and mortality rates. Salient among these are the various species of Ebola virus, which have caused recurrent outbreaks in Central Africa since the virus’s identification in 1976, as observed with the recent Ebola virus outbreaks in the Democratic Republic of the Congo (April 2022), and the Republic of Guinea (February to June 2021). Ebola viruses have had significant case fatality rates ranging from 40% to 100%. The reported number of deaths surpassed 11,000 in Guinea, Liberia and Sierra Leone during the 2014 epidemic alone in a span of two years [12]. Furthermore, zoonotic diseases may have played a considerable role in the loss of ancient civilizations, including Khmer [13] and Mayan [14] civilizations, underscoring the importance of understanding the dynamics behind spillover for preventing future emerging infectious diseases. For the period between 2010 and 2020, influenza virus infections were reported to have led to 700,000 hospitalizations annually in the USA alone [15]. In light of these recurrent and ongoing epidemics, particularly for communicable viruses, research into potential reservoir hosts has gained new importance. This applies to tentative hosts including palm civets, mink, white-tailed deer, raccoons, pangolins, porcupines, non-human primates and bats, all of whom contain cellular receptors paralogous to human virus receptors. A few among these have recently been sidelined as the potential source or intermediate hosts for the zoonotic transmission to humans [16,17], but bats continue to be investigated by numerous research groups.

Owing to the high longevity quotient recorded in bats [18], and their often-nocturnal foraging habits, mobile parasites harbored by them may have a pivotal role in pathogen maintenance or transmission. In bat roosts, bat flies have been shown to commute intraspecifically and interspecifically [19]. This is especially so given the observation that host species’ specificity may also be flexible among obligate hematophagous bat flies [20] (bat-specific dipteran ectoparasites). In a roost characterization study meant to delineate ecological factors governing the fidelity of bat–bat fly interactions, bat flies were found to be less specific in roosts with lower host species diversity [21]. A pertinent concern is whether interspecific infestation of bats by bat flies, and especially the winged species among *Streblidae,* within a given roost amounts only to transient accidental infestation. During their reproduction, female bat flies leave their hosts and deposit the third-instar larvae on roost substrates. This is followed by pupation and morphological development that may produce a viable fly within a three-week period. Subsequently, these new members mount fresh hosts. However, the female, upon larviposition, mounts a fresh host within the shortest time possible as it may not sustain prolonged free living. Herein lies the potential of bat flies as pathogen vectors between bats [22]. Among these parasites, apterous *Nycteribiidae* and brachypterous as well as flying *Streblidae* are most notable. Other arthropods found on bats include chiggers, fleas, mites (both *Acari* and ticks), bedbugs (*Cimicidae*) and bat bugs (*Polyctenidae*).

The detection of viral sequences in blood-feeding bat flies necessitates widespread inquiry into the contribution of these flies to viral epidemiology. In the establishment of an outbreak or epidemic, a vector-to-host transmission occurs first and is followed in some instances by inter-host circulation. However, as inferred from a *Drosophila* infection experiment, an additional phase may be involved. In the experiment, adult fruit flies were found to infect each other with Drosophila C virus (DCV), a positive-stranded RNA virus in the family *Discistroviridae* whose members are non-pathogenic to humans. The complete set of potential hosts for DCV is not fully understood [23]. In that experiment, it was found that up to 80% of cohoused flies acquired DCV infection from dead flies. It was further shown that viral loads in the newly infected flies corresponded to that of the donor species two days after infection. Among bat flies, vertical transmission of Ledanteviruses has been observed in Nycteribiid flies parasitizing Angolan soft-furred fruit bats (*Lissonycteris angolensis ruwenzorii*) [24]. It is therefore necessary to delineate the role of this bat fly-to-bat fly transmission phase in viral spread. For instance, the isolation of DENV from bat flies in Mexico, in a region where DENV was previously isolated from both insectivorous and frugivorous bats, portends a bat-associated life-cycle [25]. Such a cycle would indicate an adaptation of DENV to this ecological niche, with bat flies as an extra reservoir for the virus. This would be analogous to the mosquito reservoirs for DENV in forest canopies [25]. A dearth of cell culture validation of the viability of these bat-fly-derived viruses prevents any assignment of culpability to these bat flies as transmitters of DENV. With cell culture isolation, a causal association (as well as active transmission) may be inferred directly as this adheres most closely with conventional Koch postulates of pathogenesis [26]. This highlights a pressing need in virus discovery endeavors to understand the vectors, reservoirs and transmission dynamics of various zoonotic pathogen.

## 2. Diversity of Bat-Fly Interactions

Arthropod-parasitizing bats are diverse and include bedbugs (*Cimicidae*), bat flies (Diptera: *Hippoboscoidea*), ticks (*Argasidae* and *Ixodidae*) and other mites (i.e., Subclass: *Acari*). Although specific families of bat flies may occasionally be found on different bat species, both *Nycteribiidae* and *Streblidae* have one to two major hosts from whom they do not disembark (See Table 1), except during larviposition (which often lasts under 25 h, followed by mounting of the nearest host), and occasional host disturbances [27]. In some studies, individual *Myotis daubentonii* and *Megaderma lyra* have been found to host up to 21 and 17 (unpublished *M. lyra* data) bat flies respectively, but typically an estimated 1.79 Nycteribiids parasitize each host, though this may vary hugely based on an array of different factors. Grooming among bats is an additional factor that decreases the abundance of ectoparasites on bats and may be a special determinant for host and within-host specificity [28].

**Figure 1:**
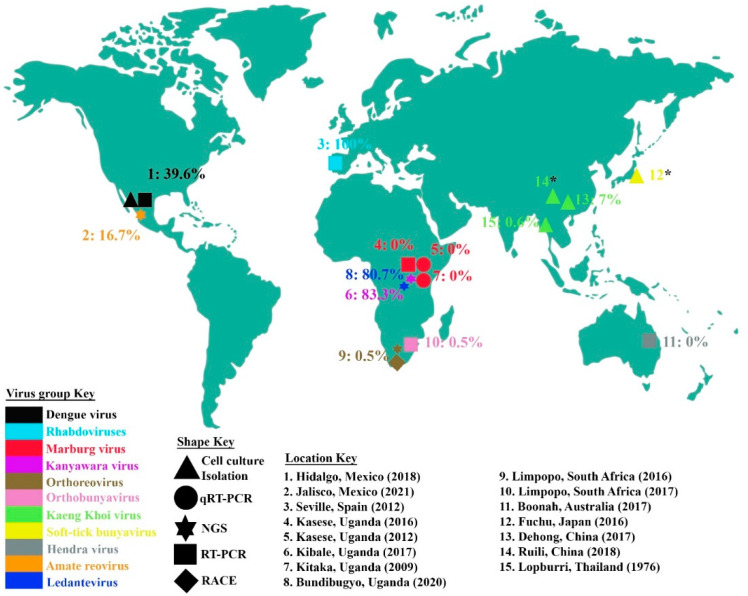
Locations from which bat ectoparasites for virus characterization were collected. The proportions of ectoparasites or ectoparasite pools found positive for the virus or virus family under study are also indicated, and virus or virus families are color-coded. The five differently shaped points show the different methods used for virus identification, including cell culture isolation, quantitative real-time PCR, reverse transcriptase PCR, next-generation sequencing and rapid amplification of cDNA ends. For the locations with an asterisk (*), the virus prevalence is indeterminate. The year for each study is also indicated.

In addition, recent dietary analysis (unpublished, carried out by authors of this study) confirms that bats sometimes consume their ectoparasites [37], and as this may be from either the self via grooming or allogrooming of other individuals, consumption of parasites may provide an additional mode of pathogen spread. In the absence of grooming, as reported among lactating female bats, bat flies were observed to reproduce rapidly and to colonize both the mother and the flying juveniles. Within the reproductive phase, bat fly infestation is favored by low immunity in the newborns and neglectful grooming among the adult bats in an effort to conserve systemic energy [38,39]. No pregnant bat flies were observed during winter hibernation of bats, an observation that highlights the responsiveness of bat fly reproduction to reproduction in their bat hosts. Ectoparasite abundance is therefore sensitive to both host physiology and environmental stimuli acting simultaneously on both the host and the parasite, although the direction of this correlation varies with host species [40]. Nevertheless, sampling effort may be considered the rate-limiting stage in identification of parasite–host interactions as identified for the streblid-bat pair [41]. A thorough investigation of reported viruses isolated from streblids and other bat ectoparasites (Table 2 and Figure 1) vis a vis the diversity of virus families known to infect metazoans shows a disparity attributable to minimal sampling effort or underreporting. It is therefore not surprising that parasites showing a wider geographical distribution are those in which there have been greater sampling efforts in research on their hosts [42]. With each successive survey of ectoparasite microbiomes, there is identification of either novel or known microbes previously believed to be endemic to alternative geographical locations. Habitat loss has been shown to reduce species diversity, and where this impacts host species, their parasites and hyperparasites meet new environmental conditions [28,43]. Termed a risky lifestyle, hyperparasitism may have arisen independently more than once [44]. The phenomenon of microbes parasitizing parasites of other organisms has been reported for bacteria, fungi, haemosporidian endoparasites and fungi [44,45,46,47].

## 3. Paramyxoviridae

Among negative-stranded RNA viruses, some paramyxoviruses have been detected in bats or bat excreta. These include Hendra virus (HeV), Nipah virus (NiV), Cedar virus (CedV) and Ghana virus (GhV) of the Henipavirus genus, whose natural hosts are Pteropodids. HeV and NiV have caused mortality of up to 90% and 100%, respectively, in humans and livestock, while CedV and GhV have shown asymptomatic to no infection in small animal models [60,61]. NiV has been responsible for human-to-human transmission in Bangladesh (and possibly India [62]) through contact with the secretions from infected individuals, pig-to-human transmission in Malaysia and Singapore, food-borne transmission in the Philippines, as well as bat-to-human transmission in Bangladesh and India. HeV outbreaks, with case fatality rates of up to 57%, have, in contrast, been reported only in Australia. In those incidents, no human-to-human transmissions have been inferred, with cases resulting from contact with infected horses and their body fluids [63]. Apart from these disease-associated reports, henipavirus sequences or antibodies against NiV have been detected in fruit bats in Thailand, China, Indonesia, Cambodia, Vietnam, India, Papua New Guinea and Malaysia. Although these provide no actionable information on prevalence and transmission potential, they point to a larger geographical area in which henipaviruses circulate within Asia and Australia.

Outside of these two continents, serological evidence and henipavirus-related RNA isolation from *Miniopterus minor*, *Coleura afra* and *Pipistrellus* spp. bats in Kenya, Ghana and the Republic of the Congo, respectively, indicate the presence of henipaviruses in those countries. A study aiming to isolate HeV from bat flies showed no positive results for the 183 bat flies collected, and no paramyxovirus has been detected in bat flies to date. This is also true for bat flies extricated from bats found to be positive for HeV virus sequences. This is possibly due to low viremia of HeV in bats. Additionally, viral shedding of HeV was determined to vary from year to year and by geographical location, in a season-independent manner, which was observed in *Pteropus alecto, P poliocephalus, P. scapulatus* and *P. conspicillatus* bat urine samples obtained from a roost in one longitudinal study [64]. In that study, it was observed that HeV prevalence rates in pteropodid bats over three years did not correlate with the periods of reported HeV spillover events. In Queensland, Australia, the highest prevalence rate for the study year was not replicated in subsequent years. Interestingly, in lower latitudes of eastern Australia, it was observed that winter peaks of HeV excretion coincided with mid- and late-gestation in *P. alecto, P. conspicillatus* and *P. poliocephalus* [65]. The gestational phase is part of a longer reproductive cycle in which these hosts are hypothesized to be more susceptible to pathogens, and bats have the longest gestation for their body size [66]. Although the sampling procedures in the above studies could neither discriminate between individual bats nor infer their morbidity or parasitic infestation due to the use of pooled urine samples, their findings highlight the existence of other environmental factors that may be responsible for spillover events. To determine these factors, bats and bat flies would need to be sampled throughout the year, in a study sensitive to both bat physiology and habitat conditions.

## 4. Rhabdoviridae

Rhabdoviruses, particularly those in the dipteran-mammal-associated *Dimarhabdovirus* supergroup, may be transmitted by an array of arthropods. These include rhabdoviruses isolated from mosquitoes, sandflies, ticks and bats [67,68,69]. Considering the high frequency of detection of viral sequences in healthy bats, it may be plausible to identify Rhabdovirus sequences isolated from nycteribiid flies in a recent bat fly study as being a sign of infection [48]. Additionally, owing to the wide host range (as they have been shown to infect both animals and plants), which encompass rhabdoviruses, the fact that all the Nycteribiid-derived sequences grouped together with rhabdovirus sequences obtained from their *Miniopterus* spp. host may support an infected arthropod hypothesis. However, the absence of rhabdovirus sequences in other species of ectoparasites found on the same bats supports a scenario where the isolated sequences are in fact endogenous elements within the Nycteribiid genome, as reported previously [70].

Within the newly constituted genus of vector-borne ledanteviruses, the earliest occurrence is associated with a painful bite from a fly within a ship returning from Western Africa to Europe. Subsequent isolations of ledanteviruses have been from both vertebrates and invertebrates, with the latest Kanyawara virus being suspected to have coevolved with its arthropod host over an unidentified period of time [24]. This may indicate a mechanism through which these viruses remain non-pathogenic in their natural hosts as well as suggest why identical ledanteviruses have not been identified in different arthropod species. Although lateral transmission has not been demonstrated, a vertical transmission regime enabled by the reproduction mode for bat flies (summarized above) has been suggested, possibly as the prime mode of maintenance within the bat flies.

## 5. Filoviridae

Members of this viral family are known to cause case fatality rates higher than 80% (above 90% for Ebola virus). Unsuccessful attempts have been made to isolate Marburg virus (MARV) from adult and nymphal *Argasidae* ticks residing in *Rousettus aegyptiacus* bat cave roosts. *Rousettus aegyptiacus* bats in the family *Pteropodidae* have been implicated as the natural reservoir for MARV. Although viral RNA sequences were isolated from the liver and spleen of approximately 2.5–3% of the bats sampled, oral swabs and ectoparasites yielded no MARV sequences [50,51]. Unlike the oral shedding of NiV observed among infected *Pteropus* spp. bats, neither oral nor excretory shedding was observed for MARV. This observation further bolsters the search for possible vectors. It has been suggested that MARV does not produce a high enough viremia for the hematophagous flies to ingest. This stems from the observation that even after experimental infection [71], and among bats found to be acutely infected with MARV (multiple organs also infected including spleen, liver, kidney, colon, ovaries and uterus), viral loads in serum are consistently low, and the bats have no symptoms of disease [52]. Hematophagous parasites may then plausibly have no viral sequences because of this low viremia.

Consistently, in an unrelated experimental infection set up, oral swabs were positive for MARV for up to four days after serum viral load had diminished to undetectable levels. Additionally, it may be possible that arthropod-specific refractory physiology hinders MARV replication in bat flies [50]. A key supportive observation is that co-parasitic arthropods found on bats have tested positively for viruses cohabiting ticks did not contain [48] (this also includes visibly engorged bat ticks [53]). It may also be argued that crevices in bat caves, from where these ticks are collected, are a degree removed from the viral reservoir (bat body system). Nevertheless, the observations that ticks are found on bats only at low frequencies, and that ticks feed less frequently than dipterans [72], suggest that the *Argasidae* family of arthropods are only incidental parasites to bats.

## 6. Peribunyaviridae and Nairoviridae

Members of the *Peribunyaviridae* family have been isolated from bat-cave-derived bedbugs (*Stricticimex parvus*) from as early as four decades ago [53]. Human activity relating to bat guano harvesting has exposed susceptible humans to Kaeng Khoi virus in caves, where additional hematophagous parasites were found. Soft ticks plucked from bat feces (a major component of bat guano) on cave floors have also been found to contain viable orthobunyaviruses [56]. These ticks, often incidentally picked up by falling bats within the cave, may have a role in the maintenance of bunyaviruses within bat roosts and bat creches. Detection of orthobunyavirus sequences in 5 of 98 ticks collected from bat-distal habitats [73] highlights ticks as potential hosts for orthobunyaviruses. Hard ticks (*Ixodid* spp.) are well-understood to be reservoirs for Crimean–Congo hemorrhagic fever virus (CCHFV, family: *Nairoviridae*) [74], but attempts to establish sustained infection of soft ticks (*Argasid* spp.) have not been successful. It may be inferred that tick genera are highly specific to the variety of bunyaviruses they may host.

Sequences for bunya-like viruses have likewise been detected in biting midges (*Culicoides impunctatus*) sampled from Scottish woodlands and thickets close to cattle herding fields [75]. No bat association is inferable from that report, and the irritation caused by these biting midges precludes the possibility of sustained infestation. However, the presence of these virus sequences highlights the scope of viral presence (infection or simple occurrence) among hematophagous flies, which may shelter in roost spaces during parts of the day.

## 7. Flaviviridae

Due to the wide range of clinical manifestations caused by flaviviruses (ranging from asymptomatic disease to fatal encephalitic and hemorrhagic disease) and the potential for their persistence, their study and surveillance is of global importance. The isolation of flaviviruses from non-arthropod hosts has primed scientific thought towards alternative potential wild and domestic reservoirs for this family of positive-stranded RNA viruses. For instance, antibodies against WNV have been detected in the serum of big brown bats in Central America [76]. Antibodies against the neurotropic Japanese Encephalitis virus were detected in 44% of serum samples in one China study [77]. Tamana virus, Koyose virus and Rio Bravo virus antibodies have also been detected in bat serum [78]. Isolation of DENV from owl ectoparasites, rodents and marsupials, as well as frugivorous and insectivorous bats, instructed the search for flavivirus sequences from bat-associated hematophagous flies. Experimental infection of bats with DENV produced low viremia, no seroconversion and absence of viral RNA in sampled tissues [79]. This observation highlights the unsuitability of bats as reservoirs for DENV.

Additionally, sequences within bat flies may well be endogenous retrotransposons. However, the isolation of DENV-2 from cell-cultured lysates provides definitive demonstration of DENV survival within these arthropods [25]. In a study from Mexico, it was observed that a majority of bat fly homogenates tested positive for DENV during the rainy season (Table 2 and Figure 1). This suggests that bat serum viral loads may be higher in that period of relative inactivity and malnutrition, due to logistical inability to forage for food [25]. Malnutrition impacts bat physiology by weakening their immunity and predisposing them to pathogen invasion. Slight-to-complete deficiency of dietary protein makes the skin and mucus membranes less competent at preventing both parasite infestation and pathogen entry [80]. Although the consensus is that DENV amplification within bats is insufficient to feed mosquitoes [81], immediate freezing of specimens after field collection has been shown to improve chances of retrieving culturable viruses. Adoption of this practice may improve the amounts of viral inoculum in C6/36 culture to better illuminate the flavivirus spectrum of specimens.

## 8. Reoviridae

Reovirus family members were previously believed to infect vertebrate hosts exclusively. This was until the isolation of viable syncytia forming virions from Nycteribiid flies taken from Egyptian fruit bats [57]. In this study, a novel orthoreovirus with 66.3% homology to bush viper virus in the RNA-dependent RNA polymerase (RdRp) sequence was identified. The virus, termed *Mahlapitsi* virus (MALV), formed a distinct clade with bush viper reovirus, Baboon orthoreovirus and Broome virus within the *orthoreovirus* genus. Although the clade was definitively distinct from bat-associated viruses, it possesses 58% homology to the bat-associated Broom virus in the RdRp amino acid sequence. All ten segments of its 23.2 kb genome were found to possess an *orthoreovirus*-genus-specific 3′ sequence. MALV did not replicate in C6/36 cells but showed appreciable replication on mammalian Vero cells, reaching peak expression at the third day. Growth in culture may not definitively delineate the possible from the unlikely hosts. However, the fact that all previously isolated reoviruses were vertebrate-associated does not overshadow the possibility that this may be a pioneering event. Additionally, as with other isolated cases of virus detection in whole-body homogenates of arthropods, it remains to be determined if these *Hippoboscoid* flies facilitate mere mechanical transmission, sustained biological transmission or both.

It is notable that the global geographical occurrence of these viruses and virus families is much broader than is depicted in Figure 1. As a representative of the *Flaviviridae* family, DENV has been reported widely, particularly in the tropical world where infections exceed 150 million annually [82]. This high incidence highlights the risk posed to humans of contracting this arthropod-borne virus. Recent projections suggest that 6.1 billion people will be at risk for DENV infection in 2080, which is much higher than the current 3 billion people at risk of infection [83]. However, no isolation or identification of DENV in bat ectoparasites has been reported outside of Mexico. Similarly, as a representative of *Filoviridae*, MARV has been reported from outbreaks in Central, West and Southern Africa as well as parts of Europe. However, even in the few studies in Uganda in which bat ectoparasites were evaluated, none were found to contain MARV. No similar studies of MARV in bat ectoparasites have been conducted other than in the cited research from Uganda. As HeV has only been identified in Australia, it is unsurprising that bat ectoparasites have not been screened for HeV in other continents. Other specific viruses for which bat ectoparasites were screened (Figure 1) have a narrower global geographical distribution, and potential reasons are discussed below. Our compilation indicates the knowledge gap in the study of bat ectoparasite viromes.

## 9. Epidemic Potential

Evidently, not all emergent viruses have caused global unrest for having surpassed vast geographical borders. Not all zoonoses (see Table 3) have been termed World Health Organization public health emergencies of international concern. Furthermore, for the viral agents with a recognized pandemic history, there is a marked variability in their case fatality rates and geographical coverage. These distinctions highlight inequality in their epidemic potential [84] (which may be defined as propensity to progress into epidemics).

Prediction of imminent outbreaks has been impossible due to the innumerable influential factors. Key among these would be ability of infected individuals (of the same species) to shed virions resulting from efficient viral replication. A phenomenon that has been observed to interfere with viral replication is the proportion of so-called defective interfering particles (DIPs). These are inevitable products of viral replication whose genetic composition is complete. They are nevertheless secreted from the infected cells upon cellular lysis and may bind to susceptible cellular receptors. This is because their structural elements are sufficient to enable receptor binding and cell entry. As with other viral groups, DIPs have been suspected to impede viral replication in arboviruses, including tick-borne encephalitis virus [114], WNV [115], Bunyamwera virus [116], RVFV [117] and Toscana virus [118]. DIPs have also been seen to promote persistence in DENV with reduced virulence [119]. 

A second significant factor in epidemic determination is genetic impairments resulting from adaptation to new hosts. Specifically, viruses often incur a viability cost that limits their survival in donor hosts after they adapt to nascent hosts [120]. An example of this kind of limitation is in poultry-adapted H7N9 and H5N1, which barely infect mammals and cannot reacclimatize to their former avian hosts [121]. While this is a factor which limits the epidemic potential of said virus in the donor host, epidemics are not an obvious result of all host switching events. In fact, most host-switching events are accompanied by maladaptive changes of the virus within the new host, and only a few cross-over events progress into epidemic status [23,120,122].

A third factor influencing epidemic potential is the abundance, distribution and motility of the nascent host [120]. Intermittent contact between geographically separated organisms increases the chance and frequency of transmission between different species. A fourth factor in which the data are equivocal relates to genetic distance from the natural host species. Although there are no favorite species pairs for interspecific virus transmission, it is generally believed that viruses may spread easily between phylogenetically closer species [120]. It has, however, been shown that susceptibility to an infection does not decrease linearly with genetic distance from the natural host. Instead, a phenomenon has been observed in which distinct clades show a uniform susceptibility to an infection, regardless of their genetic distance from the natural host [23].

## 10. Discussion

Parasitism by arthropods which only infest the host during feeding, but live and reproduce elsewhere, are well-documented phenomena among mammals [123]. In these relationships, the arthropods breed on or in close proximity to mammalian settlements in which they are nourished through periodic infestation of hosts with variable specificity. For instance, female *Anopheline* mosquitoes breeding in stagnant water pools within human settlements obtain their blood meals not only from human but also from canine, porcine, bovine, feline and hircine hosts, as shown by genetic analysis of their blood meals [124]. Rodents cohabiting in human homesteads are also hosts to both free living and obligate parasites of human beings. In a recent study assessing the ectoparasite prevalence on mammals across a habitat disturbance gradient in southwestern Madagascar, the parasitism of hosts was found to differ markedly. *Rattus rattus* sampled from human habitations hosted a higher diversity of ectoparasites than *R. rattus* sampled from both vegetated shrub-lands and degraded forests [125]. The study confirms the phenomena of shared parasites originating from humans, with an emphasis on mites. Its scope was, however, insufficient to reveal pathogen exchange between the studied hosts. It may well be coincidental that these free-living parasitic arthropods are associated with the transmission of potentially fatal pathogens, such as plasmodium. Conversely, obligate parasitic arthropods (spending most of their lives on the host) appear to have a near commensal relationship with their host. This may presumably be a result of evolutionary adaptation for the maintenance of their livelihood. The effects of these obligate parasites range from slight irritation (as with wing mites in *Myotis* bats) to lesions and dermal cysts (as with *Ascodipterans*) to paralysis [126] (caused by some members of the *Ixodes* genus of ticks).

Understandably, most of the scientific research focuses on the more severe parasite effects that may lead to mortality, with seemingly tolerable ectoparasites neglected by comparison. However, as shown by the periodic recurrence of pathogens within specific bat roosts, the role of pathogen maintenance may be a significant contribution of obligate ectoparasites. As previously mentioned, these pathogenic hyperparasites (parasites of the arthropod parasites) include bacteria, fungi and viruses. A key example is the observation that *Pseudogymnoascus destructans* infestation of ectoparasitic bat wing mites was correlated to white nose syndrome occurrence in *Myotis myotis* bats hosting these mites [127]. A consequence of this is that bat wing mites are implicated in the transmission of *P. destructans* from contaminated to naïve roosts during cave switching by infested bats. Fleas (*Siphonaptera*) are an even less studied group of bat ectoparasites [34]. Despite their lower frequency of occurrence on bats, the fact that they are shared among at least three orders of mammals, including primates, positions them as efficient vectors for pathogens. Fleas are known to transmit *Yersinia pestis* (plague) and *Ricketssia typhi* (Murine typhus) [128]. Furthermore, whilst mites are likely to require direct contact between individuals to spread, fleas can easily disperse between individuals that are not in physical contact, and may not be specific to particular mammals, meaning that they may move between species in areas utilized by multiple taxa.

It was observed that identification of a pathogen in a competent vector often restricts research in the given vector, with the effect of hampering research on alternative potential vectors [129]. This assertion is irrelevant for bat-associated viruses as no competent arthropod vector has been characterized yet. However, the difficulty of their manipulation may explain why the potential role of bat flies (and particularly within the bat-exclusive *Hippoboscoidea*) as vectors, or origins/sources of viruses, is yet to be ascertained. This challenge notwithstanding, the mobility of bat flies, as well as their parasitism of bats, means that they may offer complimentary insights into the life history of bats. Furthermore, non-pathogenic viruses simultaneously identified in ectoparasites and their bat hosts may act as markers to provide valuable clues on the nature of virus transmission between these species. A comparison of genomic features between these sequences, with the aim of determining ancestral and derived features, may reveal the mechanics and direction of the transmission.

Other species-specific reasons still limit research into the epidemiology of viruses. For instance, a number of intermediate hosts for bat-derived viruses have been proposed, including pigs for NiV and horses and dogs for HeV (as well as other susceptible livestock reared together with these [130]), and experimental infection of horses and dogs has shown a pattern of viral shedding consistent with naturally infected animals [131]. The additional observation of a higher rate of asymptomatic infection in dogs, coupled with the subtlety of symptoms when present, potentiates rapid domestic transmission to humans. However, for horses, the logistical impediment of handling them within Biosafety Level (BSL)-4 laboratories suggests why there are such few infection studies.

As obligate hematophagous parasites, the survival and reproduction of bat flies is responsive to bat health, and the tracking of changes in bat physiology informs their reproductive decisions. For instance, *Nycteribia schmidlii, Penicillidia conspicua* and *Spinturnix psi* were all found to reproduce more intensely during the gestation and nursing season of their *Minopterus schreibersii* hosts [39]. Due to this responsiveness of bat flies to bat body conditions, bat ectoparasites have been proposed as a study tool to substitute intrusive procedures on bats as a measure towards bat conservation [132]. Bat flies therefore provide a vital tool in bat health studies.

Secondary information relating to the abundance and diversity of pathogens hosted by bats may also be obtained from studies of bat flies. Due to the colonization of bat fur and other surfaces by these blood-feeding parasites and the suitability of blood as a means of pathogen dispersal, proximity alone may potentiate shared microbiomes between bat flies and bats. An example is found among bacteria isolated from both bat flies and their hosts, which may have been shared either through blood meal or mere abrasion. Among blood-bound microbes that may be transferred through blood meals into bat flies, viruses may arguably be the most interesting, due to historical outbreaks resulting from bat-associated viruses. It would also be interesting to investigate whether interactions between the virus and the host microbiota play a role in influencing host defenses against viral infection, potentially impacting virus transmission [133].

As noted above, bats host numerous viruses, some of which are pathogenic to humans. Most of these and others are maintained in bats by poorly understood mechanisms, without progression to virulence. In some studies, bats found within the same roost are shown to largely host the same repertoire of viruses. This is also true for bat roosts in which the resident bats roost in sparse gatherings, with appreciable distances between the bats. Two possible mechanisms of maintenance of these viruses may be vertical transmission to offspring, as well as horizontal transmission during grooming, allogrooming and mating. Although this may plausibly account for the larger proportion of transmission, the obligatory departure from and mounting of bats during the reproduction of bat flies summarized above may act as an additional means through which bats share and maintain pathogens [25]. However, because there are few studies on bat fly viromes, it is currently difficult to generalize on the role of bat flies as either vectors or sources of outbreak pathogens.

In the evaluation of arthropods as pathogen vectors, a key distinction has increasingly been highlighted that categorizes shuttling/transfer events as either the result of mechanical conveyance or active transmission. In the former instance, pathogens bound to the exoskeleton as an inadvertent result of physical touch or mere proximity are conveyed from one location (or host) to another. This also encompasses the more systemic but less effective transmission of imbibed/ingested pathogens that are incapable of infecting the vectors but still reside on the surface of these arthropods. On the other hand, active transmission involves the ingestion or uptake of a pathogen by a vector, in which case the pathogen infects the vector’s organs, resulting in replication of the pathogen in question. The replicated pathogen, whose titer is now presumably higher within the vector, is then transmitted from the salivary glands of the vector into the bloodstream of the host during feeding. Active transmission also encompasses the transfer of insect-specific viruses, which are not from ingestion or other uptake, but were received vertically during reproduction [134]. Due to insufficient exploration of this aspect of vector competence, it has been difficult to conclusively ascribe a vectorial role in the case of virus presence in ground bat flies (or simply homogenates) [11,24,25,48].

To verify vector status, an infection experiment would be needed in which mechanical transmission due to physical induction can be distinguished from the transmission from infected bat flies. Alternatively, upon identification of shared viral sequences from both bats and their ectoparasites, a phylogenetic study may be able to illuminate shared origins and mutations between these similar viruses and therefore justify more complex infection studies. Whilst many bat parasites are specialists and only likely to transfer pathogens between bats, some, such as fleas and ticks (though rarer), are more generalist and may transfer pathogens with other species. Overcrowded, disturbed settings offer a greater chance for parasites to move between individuals of both the same and different species, and many areas (caves, farm buildings and houses) may be in common use with multiple species in close proximity and facilitate the transfer of parasites. This potentiates another route of spillover [135]. In more disturbed habitats, and especially where bats coexist with domestic animals, there is the potential for pathogen transfer to species which may come into more direct contact with humans, thereby enabling viral transmission.

Different genomic tools have been used to investigate the viruses of bat ectoparasites. NGS-based identifications have yielded results that cannot be directly compared to the RT-PCR-based reports from different groups. This is because the criteria for positive virus identification varies by study. It is nevertheless clear that additional virus surveillance studies in bats and their ectoparasites are needed to determine the potential of these ectoparasites as vectors, and building on this to understand how stress and landscape structure exacerbates the risk of potential spillover through driving contact, and increasing viral shedding. Additionally, pan-viral/metaviromic investigations or the alternative targeted approaches used together may improve the comparability between studies on bat ectoparasite viromes.

## Figures and Tables

**Table 1:** Bat ectoparasite characteristics.

Bat Ectoparasite	Body Length	Feeding Habit	Lifespan and Mode of Reproduction	Mode of Locomotion	Refs
*Ascodipteron* (Diptera: *Streblidae*)	1–6 mm	Obligate parasitic blood feeders Male: blood feeding from the bat body surface Female: blood feeding while encysted in epidermis of host	Lifespan: Unknown Reproduction by viviparous puparity	Walking, jumping (Similar for females prior to epidermal encystment)	[22,29]
All other *Streblidae* (Diptera)	1–6 mm	Obligate parasitic blood feeders No free-feeding larval stage.	Lifespan: 130–200 days Reproduction by viviparous puparity Female must detach from host before depositing larvae on roost wall	Short flight for winged species, can fly short distances between individuals. Jumping in wingless and winged flightless Streblids.	[30,31]
*Nycteribiidae* (Diptera)	1.8–5 mm	Obligate parasitic blood feeders. Ctenidea enable attachment to hosts. No free-feeding larval stage	Lifespan: 136–190 days Reproduction by viviparous puparity Duration between egg production and hatching: 9 days 3–16 offspring in a lifetime	Rapid sliding motion through host pelage enabled by their dorsa-laterally flattened bodies. Bat individuals need to be in close proximity for Nycteribids to move between individuals ‘Swimming’ in bat fur.	[30,31]
Ticks (Ixodida: *Argasidae, Ixodidae*)	2–30 mm	Skin attachment by hypostome, and creation of feeding lesion. Obligate blood and lymph feeders	Life span: up to 20 years (varies with species) Fall off the host to deposit 200–23,000 eggs per generation	Walking, can wait on surfaces for new hosts. Jumping	[32]
Fleas (Diptera: *Siphonaptera*)	4–5 mm	Adults: obligate blood feeders Larvae: organic debris in host habitats Anautogenous	Life span: 2–8 months Oviposition both on and off host (species-dependent). Duration to hatching: 2–21 days.	Interhost: jumping between bat individuals Intrahost: walking	[31,33,34]
Mites (Acari)	0.2–2 mm	Varies widely with species, Bat-borne mites are not obligate blood feeders.	Life span: 23–1000 days	Walking, bats likely need to be in physical contact to spread between individuals	[35,36]
Bedbugs (Hemiptera: *Cimicidae*)	4–7 mm	Facultative parasites Can survive extended periods of no feeding (up to 1 year)	Life span: 6–12 months Traumatic insemination 200–500 eggs laid per cycle Duration to hatching: 7–10 days	Walking, waits on cave surfaces between feeds, easily moves between individuals	[31]
Bat bugs (*Polyctedinae*)	3–10 mm	Obligate parasites and blood feeders	Life span: unknown Pseudoplacental viviparity	Crawling, waits on cave surfaces between feeds, easily moves between bat individuals	[29,36]

The table shows bat ectoparasite characteristics including body length, feeding habits, reproduction strategy and mode of locomotion.

**Table 2:** A worldwide summary of studies on bat ectoparasite viruses.

Virus or Family	Location	Ectoparasite Family/Species/Group	Bat Species/Bat Family Evaluated	Isolation of Sequence or Live Virus	Speculations on Bat–Ectoparasite–Human Spillover	Ref
(a) Dengue virus	North America (Mexico)	Diptera: *Streblidae, Strebla wiedemanni* and *Trichobius parasiticus* **(39.6% prevalence, n = 96)**	*Desmodus rotundus*	Flavivirus sequences detected by RT-PCR of NS5 gene C6/36 cell culture. One sample positive	Role in transmission undetermined	[25]
(b) Rhabdoviruses	Europe (Spain)	Nycteribiid flies **(100% prevalence, n = 9)** Bat bugs **(0% prev, n = 3)** Bat ticks **(0% prev, n = 20)**	*Myotis daubentonii.* *Miniopterus schreibersii,*	RT-PCR targeting the rhabdovirus L gene Intracranial inoculation in mice (no virus isolation)	No inference made about transmission	[48]
	East Africa (Uganda)	Hippoboscoid (family *Nycteribiidae*), **80.7% prevalence, n = 26**	*Lissonycteris angolensis ruwenzorii*	NGS	Transmission to humans not inferred. However, vertical transmission within flies inferred due to codivergence of flies and the *ledanteviruses* present	[24]
		Nycteribiid (*Cyclopodiinae),* **83.3% prevalence, n = 6**	*Myonycteris* spp.	NGS	Incidental biting of humans by bat flies inferred to be the cause of unapparent rhabdovirus infection among Africans	[11]
	North America (Mexico)	Streblidae (*Aspidoptera phyllostomatis, Megistopoda aranea, Trichobius yunkeri)* **16.7% prevalence, n = 12**	*Pteronotus parnellii*	NGS	Streblid flies are a potent reservoir for Amate reovirus	[49]
(c) Marburg virus	East Africa (Uganda)	Argasid ticks (*Ornithodoros faini; = Carios faini, Alectorobius faini)* **(0% prevalence, n = 625)**	*Rousettus aegyptiacus*	qRT-PCR (against the matrix VP40 gene)	Ticks probably play no role in the transmission and enzootic maintenance of MARV	[50]
		*Argasidae* ticks (*Carios faini*) *Nycteribiidae* **(0% prevalence, n = 125)**	*Rousettus aegyptiacus*	RT-PCR (based on NP, VP35 and VP40 genes)	Bat flies are neither reservoirs nor vectors	[51]
		*Argasidae* ticks **(0% prevalence, n = 14 pools of 10–20)**	*Rousettus aegyptiacus*	qRT-PCR (based on NP and VP35 genes) Cultured in VeroE6 cells and subsequent IFA	Potential involvement of arthropod vectors not ruled out	[52]
(d) Bunyaviridae (Kaeng Khoi virus)	Asia (Thailand and China)	Bedbugs: *Stricticimex parvus* and *Cimex insuetus*	*Tadarida plicata*	Cytopathic effect (CPE) on cell culture and subsequent identification by neutralization test	Data implicate the bedbugs as possible vectors for KK virus	[53]
		*Eucampsipoda sundaica*	*Rousettus leschenaultii*	CPE in cell culture in BHK cells and none in C6/36 cells.	No inference made on zoonotic potential	[54]
		*Eucampsipoda sundaica*	*Rousettus leschenaultii*	CPE in VeroE6 and BHK cells.	No inference made on zoonotic potential	[55]
Soft tick bunyavirus (STBV)	(Japan)	Soft ticks *(Argas vespertilionis)-* From bat faeces	(Indeterminate)	CPE on Vero cells, NGS	No inference made on zoonotic potential	[56]
(e) Novel Orthoreovirus (MAHLV)	Africa (South Africa)	*Eucampsipoda africana* Theodor (Diptera: *Nycteribiidae*) **(<1% prevalence, n = 273)**	*Rousettus aegyptiacus*	CPE on VeroE6 cells (syncytia formation)	Pathogenicity to bats and humans yet to be determined	[57]
(f) Novel orthobunyavirus (Wolkberg virus)	(South Africa)	*Eucampsipoda africana* **(<1% prevalence, n = 273)**	*Rousettus aegyptiacus*	RT-PCR and non-characteristic CPE on VeroE6 cells, low CPE on HEK293 cells	Pathogenicity to bats and humans yet to be determined	[58]
(g) Hendra virus (HeV)	Australia	*Cyclopodia (Diptera: Nycteribiidae)*, **0% prevalence, n = 183**	*Pteropus alecto,* *Pteropus scapulatus,* *Pteropus poliocephalus*	RT-PCR targeting the HeV M gene	No role of bat flies in HeV transmission, 2–15% prevalence in flying fox reported (+ve RNA screen)	[59]

The table summarizes bat ectoparasite virome studies conducted globally with an indication of the general area (continent/sub-continent), ectoparasite family or species, method of virus isolation or identification, and inferences made on ectoparasite role in virus transmission. *Prevalence* refers to the proportion of ectoparasites or ectoparasite pools found positive for the virus family/species under study, and n is the number of ectoparasites or ectoparasite pools.

**Table 3:** Summary of published studies on viruses found in bat ectoparasites.

Viruses Pathogenic to Humans with Probable Bat Origin	Possible Bat Reservoir	References	Ectoparasites Known to be Associated with Bat Species (Family/Genera)	References
Marburg virus (MARV)	*Rousettus aegyptiacus, Rinolophus eloquens,* *Hypsignathus monstrosus,* *Miniopterus inflatus,* *Epomops franqueti*	[51,85,86,87]	*Nycteribidiae*: *Eucampsipoda Africana, Nycteribia schmidlii, Basilia robusta, Eucampsipoda hyrtlii, Dipseliopoda biannulate, Nycteribia scissa, Nycteribia hoogstraali, penicillidia fulvida, penicillidia pachymela, Basilia tenuispina, Basilia (paracyclopodia)bouvieri* *Streblidae*: *Brachytarsina allaudi, Raymondia intermedia, Raymondia seminuda, Dipseliopoda arcuata, Raymondia tauffliebi*	[88,89,90]
Nipah virus (NiV)	*Pteropus lylei* *Pteropus hypomelanus* *Pteropus vampyrus* *Pteropus giganteus*	[91,92,93]	Bat flies: *Cyclopodia dubia*, bat mites and bat bugs	[94]
Hendra virus (HeV)	*Pteropus alecto, Pteropus conspicillatus* *Pteropus scapulatus* *Pteropus poliocephalus*	[64,95,96]	*Nycteribiidae*: *Cyclopodia australis, Cyclopodia albertisii*	[97]
Menangle virus (MenV)	*Pteropus alecto,* *Pteropus conspicillatus,* *Pteropus poliocephalus,*	[98,99]	*Nycteribiidae*: *Cyclopodia australis, Cyclopodia albertisii*	[97]
Ebola virus (EBOV)	*Epomops franqueti, Hypsignathus monstrosus, Myonycteris torquata,* *Eidolon helvum,* *Epomophorus gambianus,* *Micropteropus pusillus, Mops condylurus,* *Rousettus aegyptiacus,* *Rousettus leschenaultii*	[100,101]	*Eucampsipoda Africana, Nycteribia schmidlii, Basilia robusta, Eucampsipoda hyrtlii, Dipseliopoda biannulate, Basilia tenuispina* *Brachytarsina allaudi, Dipseliopoda arcuate, Cyclopodia greeffi, penicillidia fulvida, Dipseliopoda setosa*	[88,89,90]
Australian bat lyssavirus (ABLV)	*Pteropus* spp., *Saccolaimus* spp., *Macroderma* spp., *Hipposideros* spp., *Chaerophon* spp., *Tadarida* spp., *Chalinolobus* spp., *Vespedalus* spp.,	[102,103,104]	*Nycteribiidae*: *Tripselia aequisetosa, penicillidia fulvida, penicillidia pachymela, Eucampsipoda africana, Penicilidia fulvida, Nycteribia schmidlii, Basilia ansifera* *Streblidae*: *Raymondia allisoni, Raymondia alulata, Raymondia aspera, Raymondia brachyphysa, Raymondia hardyi, Raymondia huberi, Raymondia intermedia, Raymondia pagodarum, Raymondia seminuda, Raymondia setiloba, Raymondia simplex, Raymondia waterstoni, Raymondia huberi, Brachytarsina allaudi, Ascodipteron jonesi, Nycteribosca alluaudi*	[88,89,90]
SARS-CoV	*Rhinolophus sinicus, Rhinolophus ferrumequinum*	[105]	*Nycteribiidae* spp., *Streblidae* spp.	*(unpublished data)*
MERS-CoV	*Pipistrellus abramus, Neoromicia zuluensis*	[106,107,108]	*Basilia majuscula, Nycteribia* spp.	[109]
SARS-CoV-2 (BatCoV RaTG13)—96.2% sequence identity.	*Rhinolophus affinis*	[110,111]	Ixodid bat ticks, *Nycteribiinae,* *Brachytarsininae*	[112,113]

The table shows viruses of probable bat origin, the bat species considered a tentative natural reservoir and the bat ectoparasite species known to associate with the listed bat species.

## Data Availability

Not applicable.
